# Supplementary material for: Bottled aqua incognita: microbiota assembly and dissolved organic matter diversity in natural mineral waters
Source: Microbiome. 2017 Sep 22;5:126. doi: 10.1186/s40168-017-0344-9 (PMC5610417; doi:10.1186/s40168-017-0344-9)
Supplement: Supplementary file 2 — Supplementary Information. (PDF 10383 kb) [file 40168_2017_344_MOESM2_ESM.pdf]

Additional file 2

Bottled aqua incognita:

Microbiota assembly and dissolved organic matter diversity in natural mineral waters

Celine C. Lesaulnier, Craig W. Herbold, Claus Pelikan, David Berry, Cédric Gérard, Xavier Le Coz, Sophie Gagnot, Jutta Niggemann, Thorsten Dittmar, Gabriel A. Singer, and Alexander Loy

Supplementary RESULTS AND DISCUSSION

Limitations of 16S rRNA gene-based microbiological surveys

16S rRNA gene-based sequencing approaches have their limitations in assessing microbial community composition and related safety aspects [1]. Most importantly, the conserved 16S rRNA molecule has insufficient phylogenetic resolution to differentiate between virulent and harmless environmental strains [2] and in some cases not even between different species or genera [3]. 16S rRNA gene sequences affiliated with pathogen-containing taxa such as the *Enterobacteriaceae* or *Pseudomonadaceae* (Supplementary Figure S3) are frequently detected in drinking water and as such not useful to assess safety risks without further contextual information [4-6].

Limitations of FT-ICR-MS analysis

The links between DOM turnover and growing bacteria established in this study are not necessarily directly causal and presumably apply mostly to semi-labile to more refractory molecules because the most labile DOM compounds might not have been adequately captured by FT-ICR-MS analysis [7] nor are they likely to exist in detectable concentrations in aged, subsurface-derived waters. Dissolved free amino acids, nucleic acids, fatty acids, and carbohydrates in nutrient-poor waters are rapidly consumed by microorganisms [8] and thus may be below the detection limit. Furthermore, our DOM extraction discriminates against mobile, highly polar compounds and the mass-spectrometric analysis is limited to masses >150 Da; C1 compounds are thus not detected.

Dissolved organic matter composition and dynamics in Water 1 and Water 2

The following examples highlight differences in DOM composition of Water 1 and Water 2. While highly unsaturated phenolic compounds comprised the bulk of the overall spectrum intensity for both waters, a larger fraction of compounds in this molecular group belonged to the oxygen-rich subgroup in Water 2. With regard to elemental composition, Water 1 samples were characterized by a distinct cluster of sulfur-containing molecules in the unsaturated/aromatic region of van Krevelen space, which displays individual sum formulas in a 2-D plot defined by O:C (oxygen richness) and H:C (saturation) ratios (Supplementary Figure S4). Across all samples, Water 1 had more sulfur- and phosphorous-containing molecules, while Water 2 had more nitrogen-containing molecules

(Supplementary Figure S4, Supplementary Table S8). The two waters also differed in mean molecular masses: polyphenolic (481.2 vs. 289.6 Da), highly unsaturated phenolic (473.9 vs. 404.7 Da), and unsaturated aliphatic compounds (417.5 vs. 350.8 Da) all had on average much larger molecular masses in Water 1 than in Water 2.

During storage DOM showed an overall increase of oxygen-rich unsaturated and highly unsaturated compounds and a concomitant decrease of oxygen-poor unsaturated compounds (Figure 4, Supplementary Figure S5). Formulas that correlated strongly negatively or positively with the main change in DOM composition – and thus are at opposed ends in a gradient of likely decreasing and increasing compounds – occupied clearly contrasting regions in van Krevelen space, differed in dominant molecular groups, and had different average molecular mass (Supplementary Figure S5). In both waters, highly unsaturated O-rich phenolic and aromatic formulas increased in relative abundance at the cost of O-poor unsaturated and aliphatic compounds.

REFERENCES

1. Ceuppens S, Li D, Uyttendaele M, Renault P, Ross P, Van Ranst M, Cocolin L, Donaghy J. Molecular Methods in Food Safety Microbiology: Interpretation and Implications of Nucleic Acid Detection. Compr Rev Food Sci F. 2014;13:551-577.
2. Wirth T, Falush D, Lan R, Colles F, Mensa P, Wieler LH, Karch H, Reeves PR, Maiden MC, Ochman H *et al*. Sex and virulence in Escherichia coli: an evolutionary perspective. Mol Microbiol. 2006;60:1136-1151.
3. Fukushima M, Kakinuma K, Kawaguchi R. Phylogenetic analysis of Salmonella, Shigella, and Escherichia coli strains on the basis of the gyrB gene sequence. J Clin Microbiol. 2002;40:2779-2785.
4. Roeselers G, Coolen J, van der Wielen PW, Jaspers MC, Atsma A, de Graaf B, Schuren F. Microbial biogeography of drinking water: patterns in phylogenetic diversity across space and time. Environ Microbiol. 2015;17:2505-2514.
5. Franca L, Lopez-Lopez A, Rossello-Mora R, da Costa MS. Microbial diversity and dynamics of a groundwater and a still bottled natural mineral water. Environ Microbiol. 2015;17:577-593.
6. van der Wielen PW, Heijnen L, van der Kooij D. Pyrosequence analysis of the hsp65 genes of nontuberculous mycobacterium communities in unchlorinated drinking water in the Netherlands. Appl Environ Microbiol. 2013;79:6160-6166.
7. Osterholz H, Singer G, Wemheuer B, Daniel R, Simon M, Niggemann J, Dittmar T. Deciphering associations between dissolved organic molecules and bacterial communities in a pelagic marine system. ISME J. 2016;10:1717-1730.
8. Egli T. How to live at very low substrate concentration. Water Res. 2010;44:4826-4837.

Supplementary FIGURES

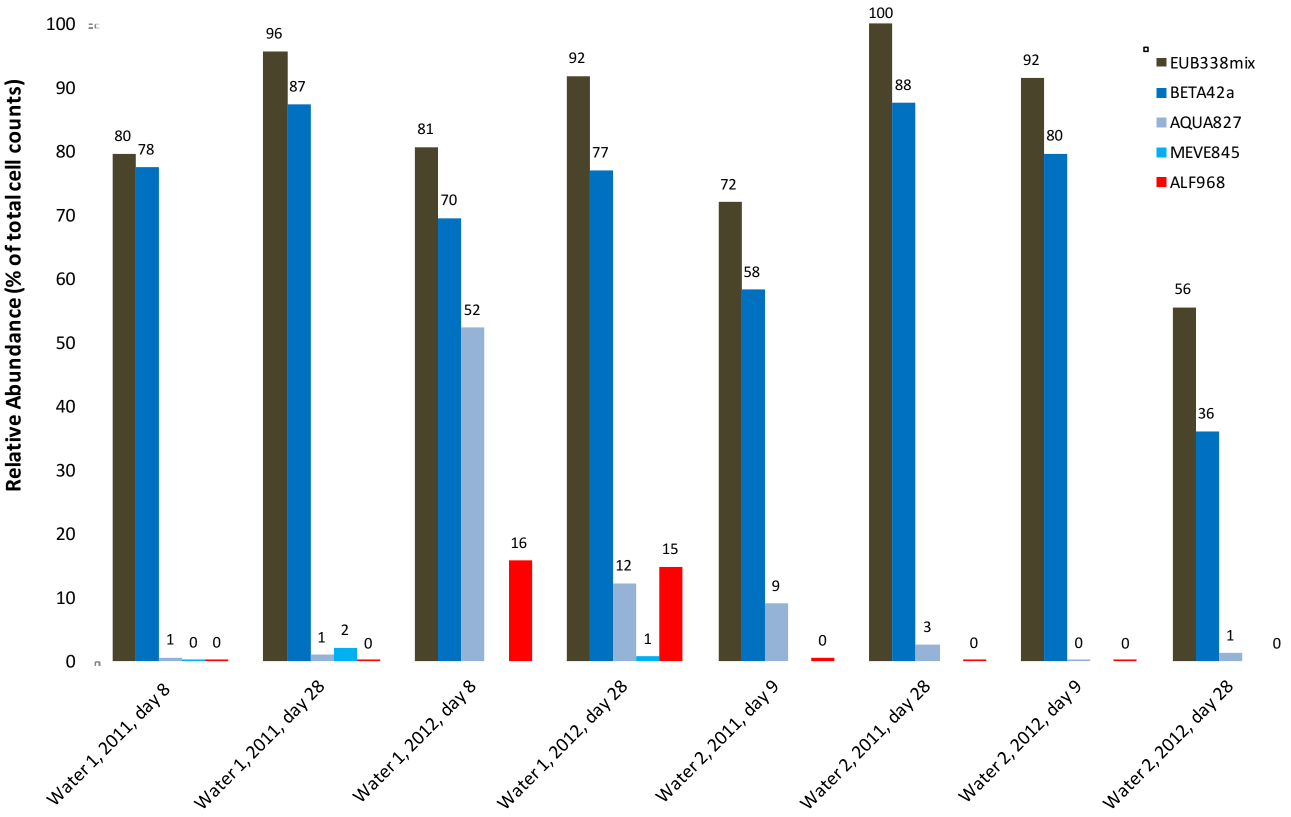

Figure S1. Relative abundances of selected bacterial groups in plankton samples from bottled water 1 and 2 as analyzed by quantitative fluorescence *in situ* hybridization. The following mono-labelled probes were applied on samples from selected days after bottling in the years 2011 and 2012: EUB338mix for *Bacteria*, ARCH915 for *Archaea*, ALF968 for *Alphaproteobacteria*, BET42a for *Betaproteobacteria*, AQUA827 for genus *Aquabacterium*, MEVE845 for genus *Methyloversatilis*, and HGC69a for *Actinobacteria. Archaea* and *Actinobacteria* were not detected by FISH. No cell showed a signal with probes EUB338mix and ARCH915 at day 1 after bottling.

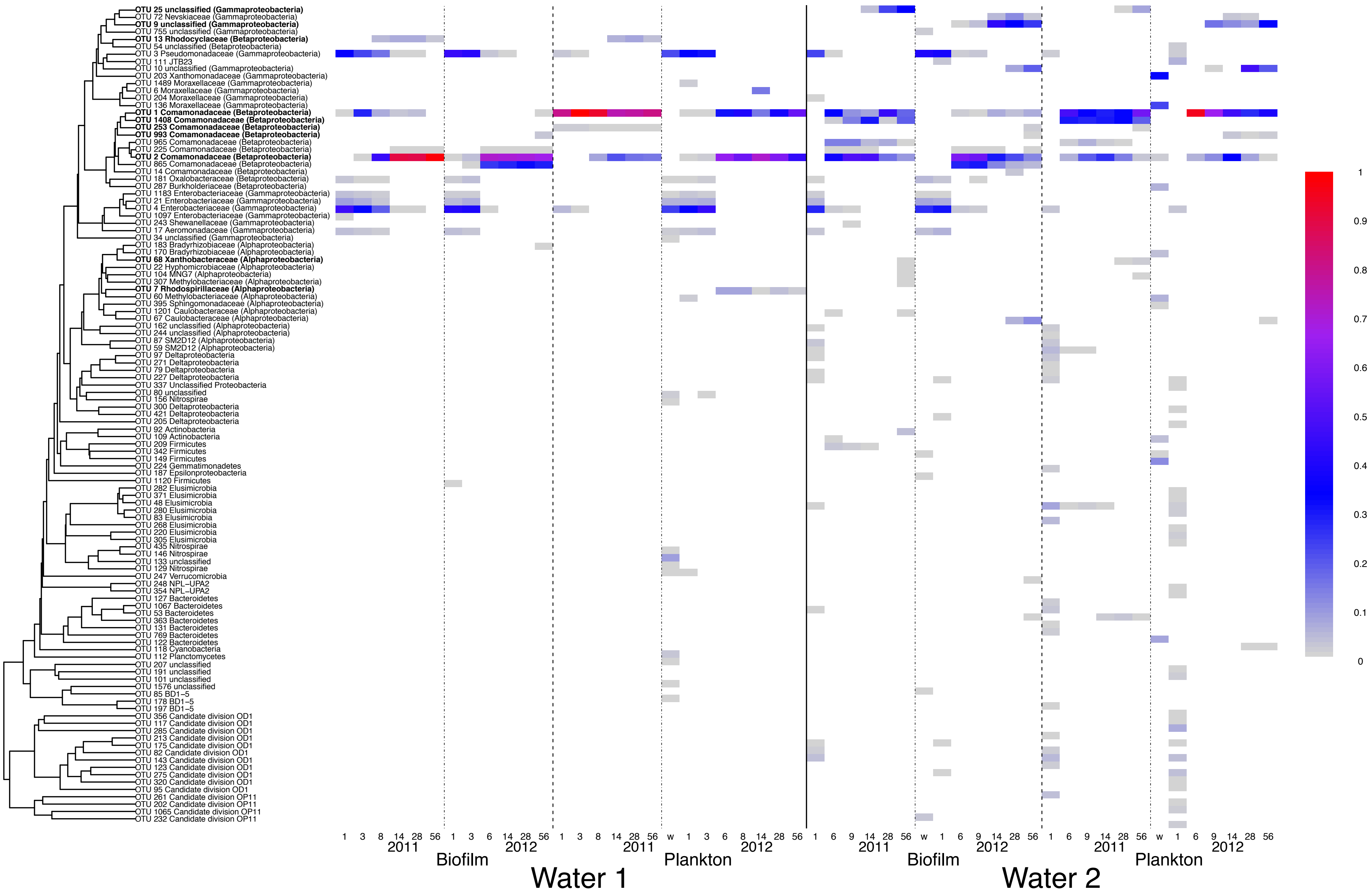
OTU 25 unclassified (Gammaproteobacteria)
OTU 72 Nevskiaceae (Gammaproteobacteria)
OTU 9 unclassified (Gammaproteobacteria)
OTU 755 unclassified (Gammaproteobacteria)
OTU 13 Rhodocyclaceae (Betaproteobacteria)
OTU 54 unclassified (Betaproteobacteria)
OTU 3 Pseudomonadaceae (Gammaproteobacteria)
OTU 111 JTB23
OTU 10 unclassified (Gammaproteobacteria)
OTU 203 Xanthomonadaceae (Gammaproteobacteria)
OTU 1489 Moraxellaceae (Gammaproteobacteria)
OTU 6 Moraxellaceae (Gammaproteobacteria)
OTU 204 Moraxellaceae (Gammaproteobacteria)
OTU 136 Moraxellaceae (Gammaproteobacteria)
OTU 1 Comamonadaceae (Betaproteobacteria)
OTU 1408 Comamonadaceae (Betaproteobacteria)
OTU 253 Comamonadaceae (Betaproteobacteria)
OTU 993 Comamonadaceae (Betaproteobacteria)
OTU 965 Comamonadaceae (Betaproteobacteria)
OTU 225 Comamonadaceae (Betaproteobacteria)
OTU 2 Comamonadaceae (Betaproteobacteria)
OTU 865 Comamonadaceae (Betaproteobacteria)
OTU 14 Comamonadaceae (Betaproteobacteria)
OTU 181 Oxalobacteraceae (Betaproteobacteria)
OTU 287 Burkholderiaceae (Betaproteobacteria)
OTU 1183 Enterobacteriaceae (Gammaproteobacteria)
OTU 21 Enterobacteriaceae (Gammaproteobacteria)
OTU 4 Enterobacteriaceae (Gammaproteobacteria)
OTU 1097 Enterobacteriaceae (Gammaproteobacteria)
OTU 243 Shewanellaceae (Gammaproteobacteria)
OTU 17 Aeromonadaceae (Gammaproteobacteria)
OTU 34 unclassified (Gammaproteobacteria)
OTU 183 Bradyrhizobiaceae (Alphaproteobacteria)
OTU 170 Bradyrhizobiaceae (Alphaproteobacteria)
OTU 68 Xanthobacteraceae (Alphaproteobacteria)
OTU 22 Hyphomicrobiaceae (Alphaproteobacteria)
OTU 104 MNG7 (Alphaproteobacteria)
OTU 307 Methylobacteriaceae (Alphaproteobacteria)
OTU 7 Rhodospirillaceae (Alphaproteobacteria)
OTU 60 Methylobacteriaceae (Alphaproteobacteria)
OTU 395 Sphingomonadaceae (Alphaproteobacteria)
OTU 1201 Caulobacteraceae (Alphaproteobacteria)
OTU 67 Caulobacteraceae (Alphaproteobacteria)
OTU 162 unclassified (Alphaproteobacteria)
OTU 244 unclassified (Alphaproteobacteria)
OTU 87 SM2D12 (Alphaproteobacteria)
OTU 59 SM2D12 (Alphaproteobacteria)
OTU 97 Deltaproteobacteria
OTU 271 Deltaproteobacteria
OTU 79 Deltaproteobacteria
OTU 227 Deltaproteobacteria
OTU 337 Unclassified Proteobacteria
OTU 80 unclassified
OTU 156 Nitrospirae
OTU 300 Deltaproteobacteria
OTU 421 Deltaproteobacteria
OTU 205 Deltaproteobacteria
OTU 92 Actinobacteria
OTU 109 Actinobacteria
OTU 209 Firmicutes
OTU 342 Firmicutes
OTU 149 Firmicutes
OTU 224 Gemmatimonadetes
OTU 187 Epsilonproteobacteria
OTU 1120 Firmicutes
OTU 282 Elusimicrobia
OTU 371 Elusimicrobia
OTU 48 Elusimicrobia
OTU 280 Elusimicrobia
OTU 83 Elusimicrobia
OTU 268 Elusimicrobia
OTU 220 Elusimicrobia
OTU 305 Elusimicrobia
OTU 435 Nitrospirae
OTU 146 Nitrospirae
OTU 133 unclassified
OTU 129 Nitrospirae
OTU 247 Verrucomicrobia
OTU 248 NPL–UPA2
OTU 354 NPL–UPA2
OTU 127 Bacteroidetes
OTU 1067 Bacteroidetes
OTU 53 Bacteroidetes
OTU 363 Bacteroidetes
OTU 131 Bacteroidetes
OTU 769 Bacteroidetes
OTU 122 Bacteroidetes
OTU 118 Cyanobacteria
OTU 112 Planctomycetes
OTU 207 unclassified
OTU 191 unclassified
OTU 101 unclassified
OTU 1576 unclassified
OTU 85 BD1–5
OTU 178 BD1–5
OTU 197 BD1–5
OTU 356 Candidate division OD1
OTU 117 Candidate division OD1
OTU 285 Candidate division OD1
OTU 213 Candidate division OD1
OTU 175 Candidate division OD1
OTU 82 Candidate division OD1
OTU 143 Candidate division OD1
OTU 123 Candidate division OD1
OTU 275 Candidate division OD1
OTU 320 Candidate division OD1
OTU 95 Candidate division OD1
OTU 261 Candidate division OP11
OTU 202 Candidate division OP11
OTU 1065 Candidate division OP11
OTU 232 Candidate division OP11
1 3 8 14 28 56
2011
1 3 6 14 28 56
2012
Biofilm
1 3 8 14 28 56
2011
w 1 3 6 8 14 28 56
2012
Plankton
Water 1
1 6 9 14 28 56
2011
w 1 6 9 14 28 56
2012
Biofilm
1 6 9 14 28 56
2011
w 1 6 9 14 28 56
2012
Plankton
Water 2
1
0.9
0.8
0.7
0.6
0.5
0.4
0.3
0.2
0.1
0

Figure S2. Relative changes in the composition of species-level OTUs in natural mineral water 1 and 2 after bottling. Temporal changes in bacterial community composition in the water (plankton) and on the inner bottle wall (biofilm) during experiments in the years 2011 and 2012 were analyzed by 16S rRNA gene amplicon sequencing. The heatmap shows only OTUs with >1% mean (across replicates) relative abundance in at least one sample. OTUs are arranged using complete-linkage clustering on pairwise distances according to Kimura's 2-parameter model. 'Growing' OTUs are indicated in bold. Numbers at the bottom of the heatmap indicate days after bottling. W, well water.

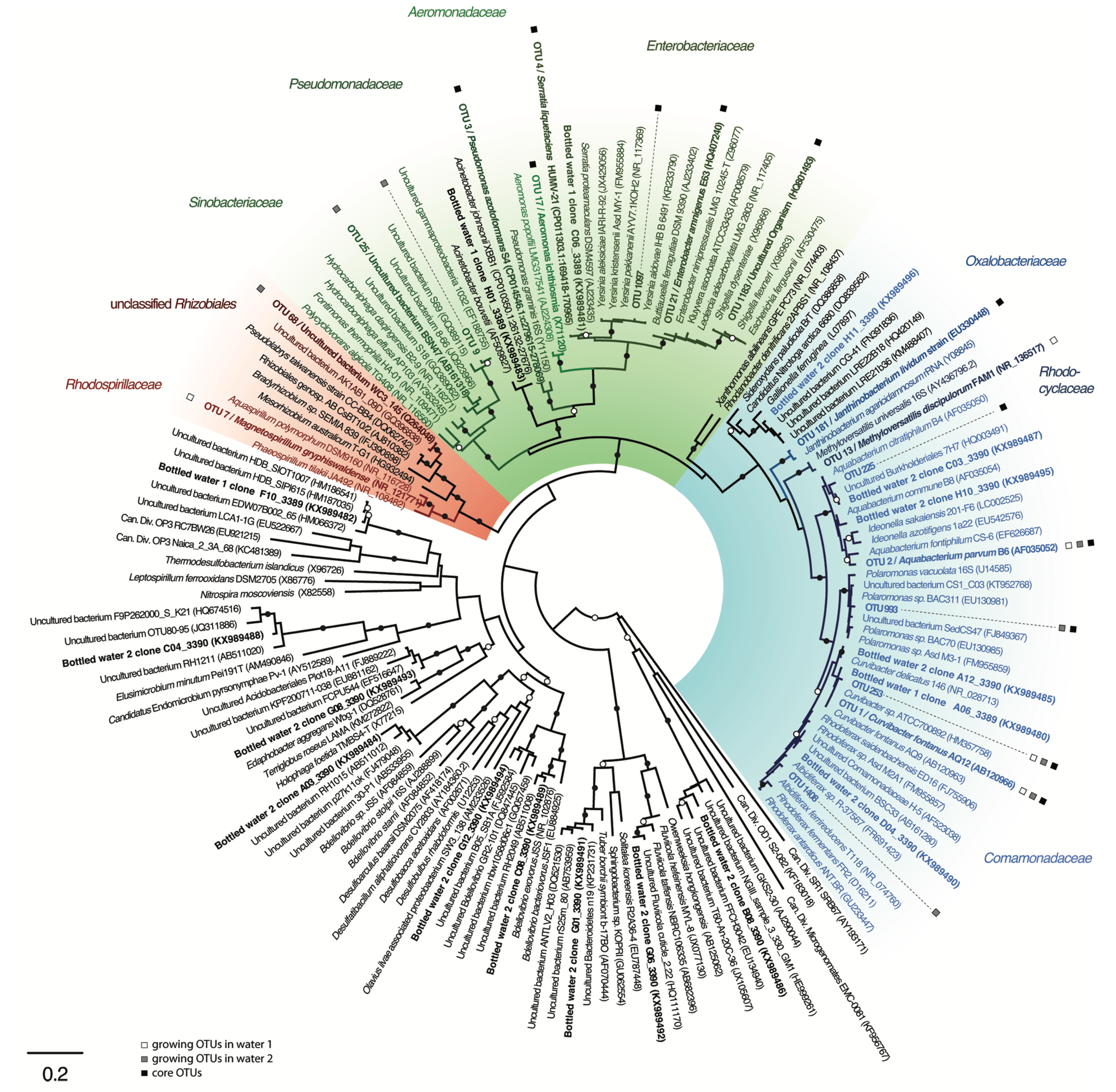

Supplementary Figure S3. 16S rRNA gene tree showing the affiliation of core and growing bottled water bacteria. Near full-length 16S rRNA gene sequences and representative reads of 454-derived OTUs that were identified as core and/or growing OTUs were used for phylogenetic reconstruction. Core and/or growing OTUs and near full-length sequences recovered in this study are shown in bold. White and grey squares indicate OTUs growing in Water 1 and Water 2, respectively. Black squares indicate core OTUs. The bar indicates 2% estimated sequence divergence. Closed circles indicate >95% RAxML bootstrap support and >0.99 Phylobayes posterior probability, respectively. Open circles indicate 70-95% RAxML bootstrap support and 0.90-0.99 Phylobayes posterior probability, respectively. *Alpha-*, *Beta-*, and *Gammaproteobacteria* are shaded in red, blue, and green, respectively.

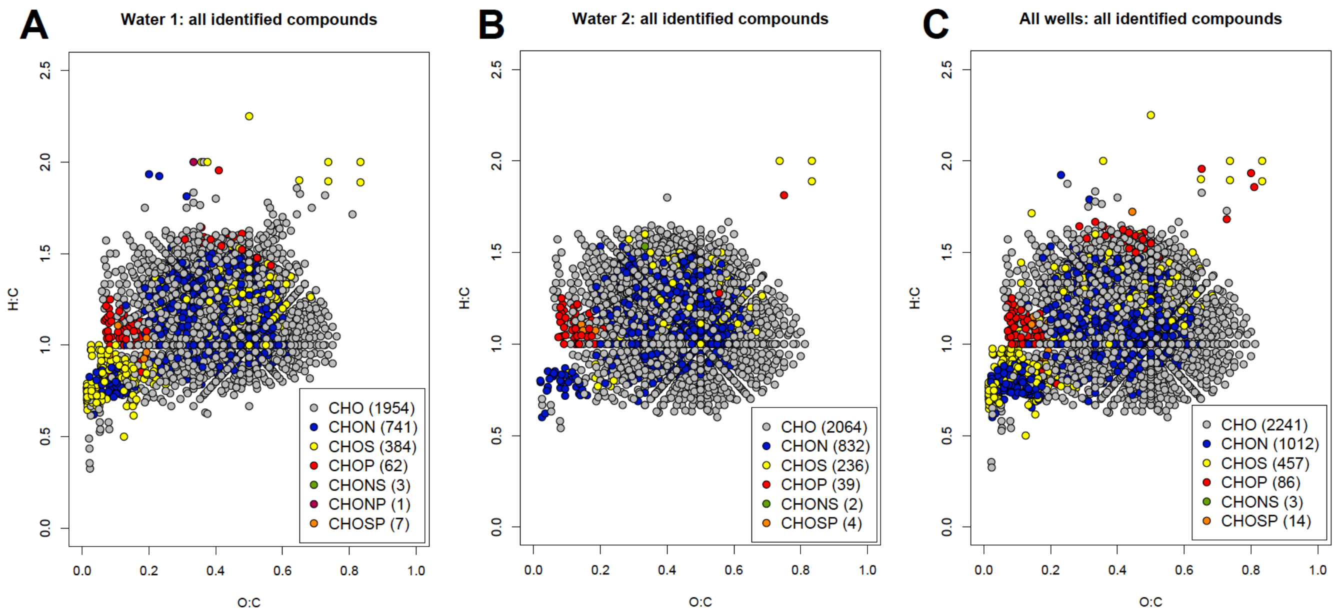

Figure S4. Elemental composition of dissolved organic matter in bottled waters. Van Krevelen plots of all 3152, 3177, and 3813 chemical sum formulas identified by FT-ICR-MS in Water 1 and Water 2 (**A, B**, time course experiment samples) and all well and bottled waters at day 1 after bottling (**C**, 'diversity study'), respectively. Each dot represents one sum formula, dot location informs about oxygen richness and saturation, dot color shows elemental composition. Dots are plotted in random order to avoid plotting bias.

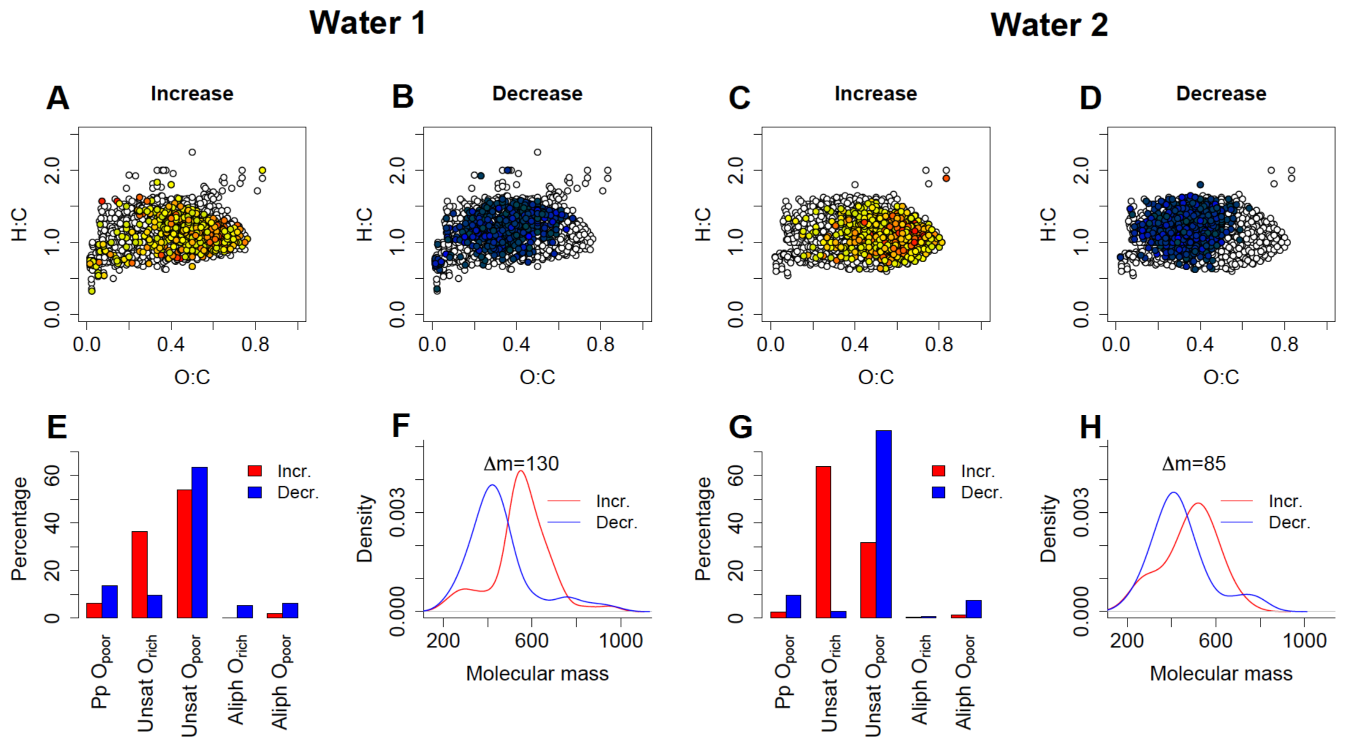

Figure S5. DOM fractions showing most pronounced temporal change in natural mineral water 1 and 2 during 56 days of storage after bottling. (A-D) For each water, two sets of formulae with very low/high correlation coefficients (<20% and >80% quantiles) with the major gradient of compositional change of DOM (PC 1) are juxtaposed in van Krevelen space. These two formula sets describe two pools of compounds likely decreasing and increasing during incubation. Color coding is analogous to Figure 4 E and F. **(E, G)** Dominant molecular groups for each of the two formula sets (Pp O_{poor}: O-poor polyphenols, Unsat O_{rich}: highly unsaturated O-rich phenolic and aromatic compounds, Unsat O_{poor}: highly unsaturated O-poor phenolic and aromatic compounds, Aliph O_{rich}: unsaturated aliphatic O-rich compounds, Aliph O_{poor}: unsaturated aliphatic O-poor compounds; see Supplementary Table S7 for definition). **(F, H)** Distribution of molecular weight for the two formula sets.

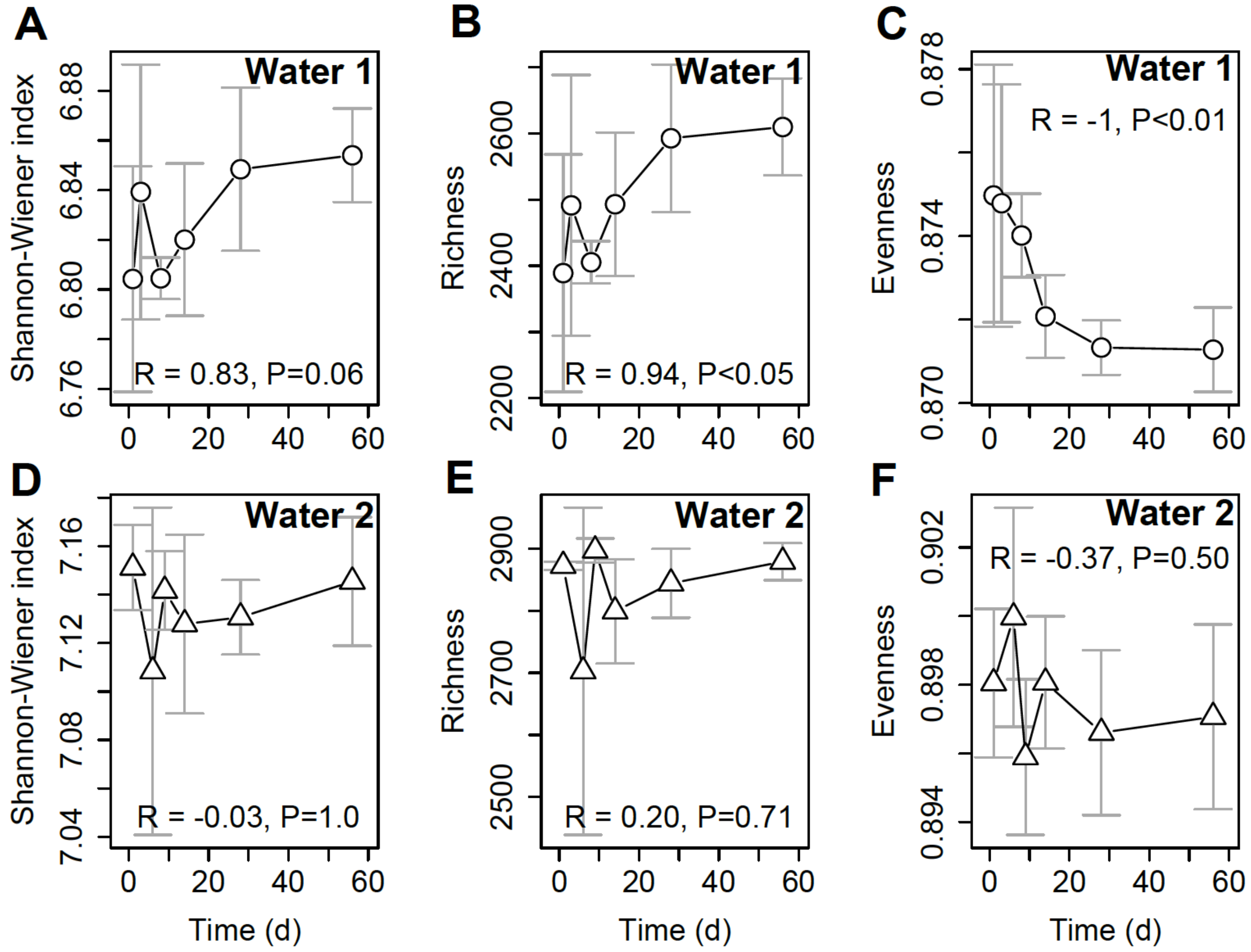

Figure S6. Changes in chemical diversity in natural mineral water 1 and 2 during 56 days of storage after bottling. (A, D) DOM diversity is given as Shannon-Wiener index, which can be partitioned into **(B, E)** richness, i.e. the number of different molecule formulas, and **(C, F)** evenness, i.e. the inverse of dominance. Data are means (±SD) for each time point (n=3).

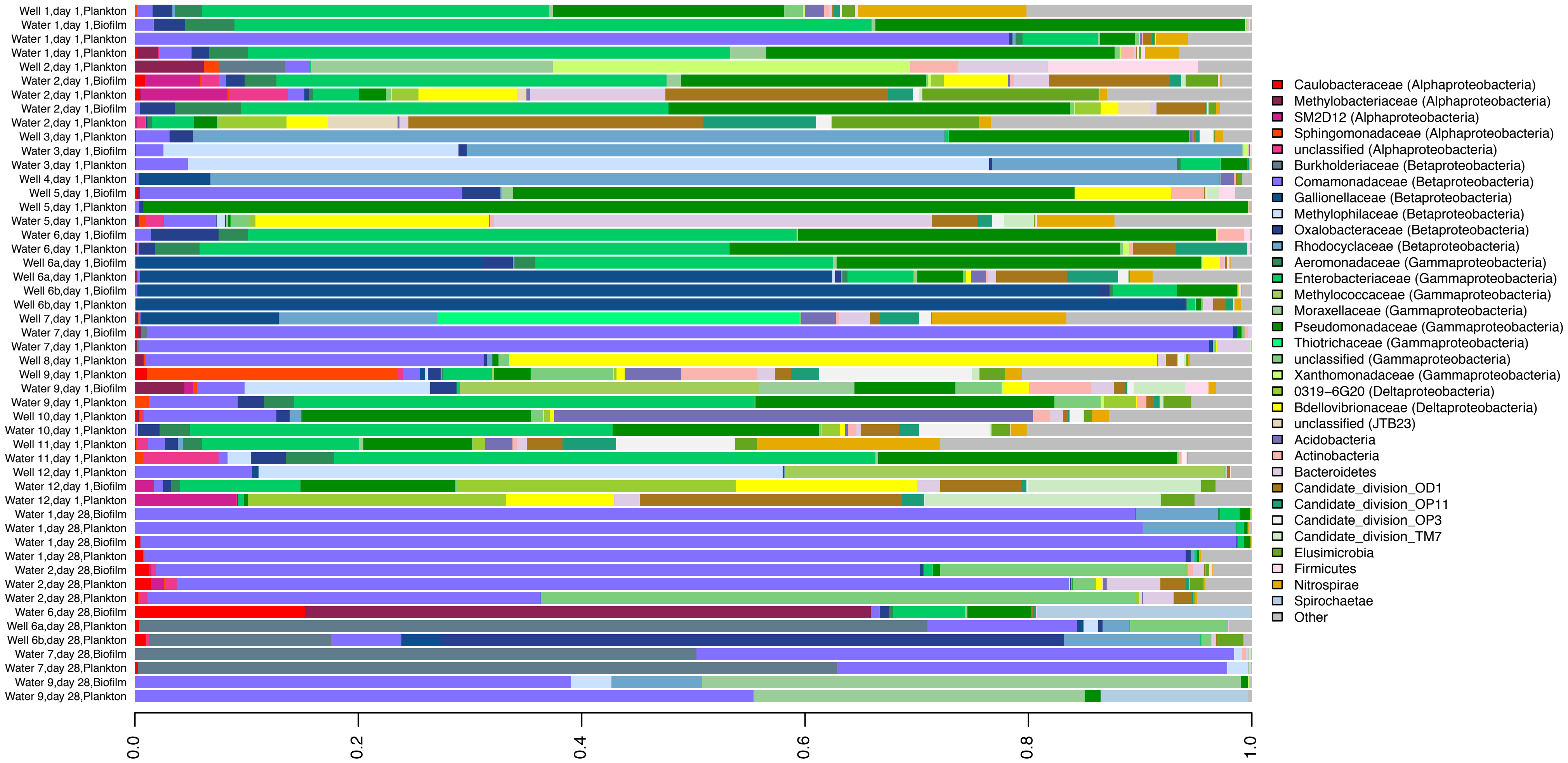
Well 1,day 1,Plankton
Water 1,day 1,Biofilm
Water 1,day 1,Plankton
Water 1,day 1,Plankton
Well 2,day 1,Plankton
Water 2,day 1,Biofilm
Water 2,day 1,Plankton
Water 2,day 1,Biofilm
Water 2,day 1,Plankton
Well 3,day 1,Plankton
Water 3,day 1,Biofilm
Water 3,day 1,Plankton
Well 4,day 1,Plankton
Well 5,day 1,Biofilm
Well 5,day 1,Plankton
Water 5,day 1,Plankton
Water 6,day 1,Biofilm
Water 6,day 1,Plankton
Well 6a,day 1,Biofilm
Well 6a,day 1,Plankton
Well 6b,day 1,Biofilm
Well 6b,day 1,Plankton
Well 7,day 1,Plankton
Water 7,day 1,Biofilm
Water 7,day 1,Plankton
Well 8,day 1,Plankton
Well 9,day 1,Plankton
Water 9,day 1,Biofilm
Water 9,day 1,Plankton
Well 10,day 1,Plankton
Water 10,day 1,Plankton
Well 11,day 1,Plankton
Water 11,day 1,Plankton
Well 12,day 1,Plankton
Water 12,day 1,Biofilm
Water 12,day 1,Plankton
Water 1,day 28,Biofilm
Water 1,day 28,Plankton
Water 1,day 28,Biofilm
Water 1,day 28,Plankton
Water 2,day 28,Biofilm
Water 2,day 28,Plankton
Water 2,day 28,Plankton
Water 6,day 28,Biofilm
Well 6a,day 28,Plankton
Well 6b,day 28,Plankton
Water 7,day 28,Biofilm
Water 7,day 28,Plankton
Water 9,day 28,Biofilm
Water 9,day 28,Plankton
0.0
0.2
0.4
0.6
0.8
1.0
Caulobacteraceae (Alphaproteobacteria)
Methylobacteriaceae (Alphaproteobacteria)
SM2D12 (Alphaproteobacteria)
Sphingomonadaceae (Alphaproteobacteria)
unclassified (Alphaproteobacteria)
Burkholderiaceae (Betaproteobacteria)
Comamonadaceae (Betaproteobacteria)
Gallionellaceae (Betaproteobacteria)
Methylophilaceae (Betaproteobacteria)
Oxalobacteraceae (Betaproteobacteria)
Rhodocyclaceae (Betaproteobacteria)
Aeromonadaceae (Gammaproteobacteria)
Enterobacteriaceae (Gammaproteobacteria)
Methylococcaceae (Gammaproteobacteria)
Moraxellaceae (Gammaproteobacteria)
Pseudomonadaceae (Gammaproteobacteria)
Thiotrichaceae (Gammaproteobacteria)
unclassified (Gammaproteobacteria)
Xanthomonadaceae (Gammaproteobacteria)
0319−6G20 (Deltaproteobacteria)
Bdellovibrionaceae (Deltaproteobacteria)
unclassified (JTB23)
Acidobacteria
Actinobacteria
Bacteroidetes
Candidate_division_OD1
Candidate_division_OP11
Candidate_division_OP3
Candidate_division_TM7
Elusimicrobia
Firmicutes
Nitrospirae
Spirochaetae
Other

Figure S7. Bacterial community composition in all well and bottled water samples at day 1 and 28 after filling. Differences in bacterial community composition in the water (plankton) and on the inner PET bottle wall (biofilm) during experiments in the years 2011 and 2012 were analyzed by 16S rRNA gene amplicon sequencing. Bar chart only shows phyla with >5% related abundance in at least one sample. *Proteobacteria* are shown as individual families.

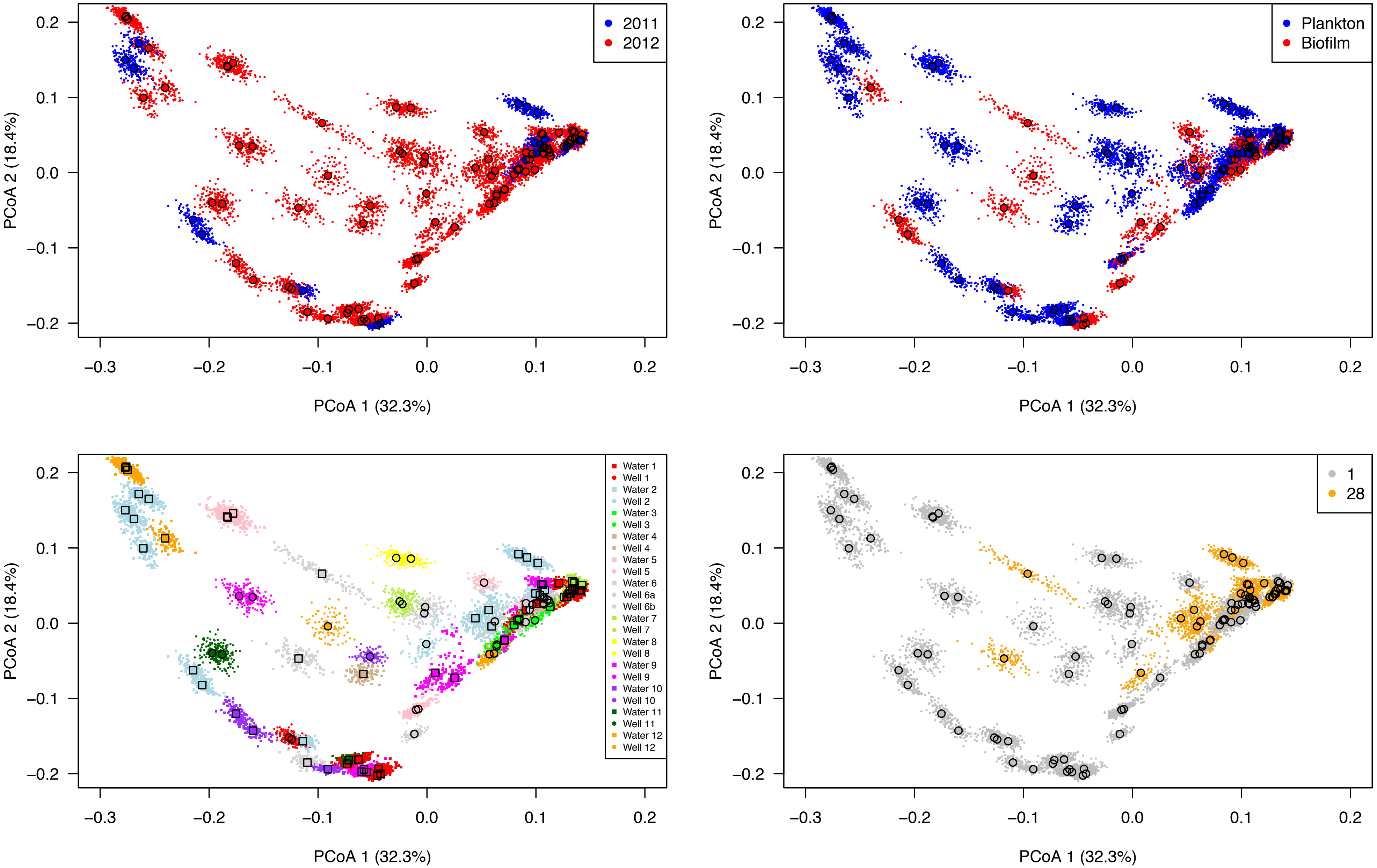
2011
2012
Plankton
Biofilm
Water 1
Well 1
Water 2
Well 2
Water 3
Well 3
Water 4
Well 4
Water 5
Well 5
Water 6
Well 6a
Well 6b
Water 7
Well 7
Water 8
Well 8
Water 9
Well 9
Water 10
Well 10
Water 11
Well 11
Water 12
Well 12
1
28
PCoA 1 (32.3%)
PCoA 2 (18.4%)

Figure S8. Microbiota beta-diversity in all well and bottled water samples. PCoA analysis based on weighted UniFrac distances calculated from bacterial 16S rRNA gene amplicon data. Each larger circle indicates the microbiota of an individual, replicate water sample. *In-silico* dataset re-sampling is visualized in PCoA plots as smaller circles. Each panel shows the same PCoA plot with individual coloring according to the year of sampling (year 2011 vs year 2012), type of microbiota (plankton vs biofilm), type of water (n = 12, well water vs bottled water), and days after bottling (1 vs 28).

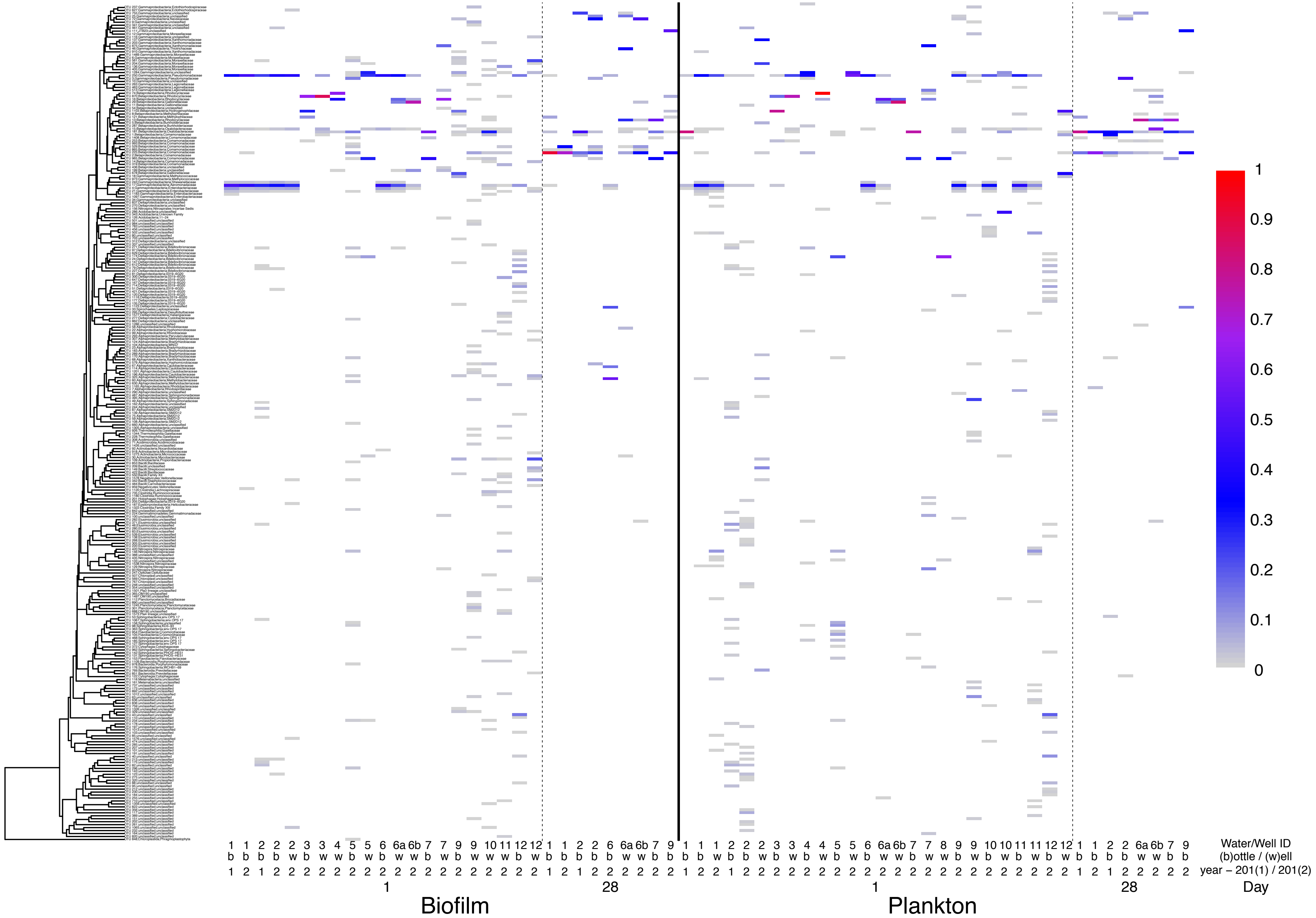

1
0.9
0.8
0.7
0.6
0.5
0.4
0.3
0.2
0.1
0
Water/Well ID
(b)ottle / (w)ell
year – 201(1) / 201(2)
Day
1
28
1
28
Biofilm
Plankton

Figure S9. Composition of species-level OTUs in all well and bottled water samples at day 1 and 28 after filling. Differences in bacterial community composition in the water (plankton) and on the inner PET bottle wall (biofilm) during experiments in the years 2011 and 2012 were analyzed by 16S rRNA gene amplicon sequencing. The heatmap shows only OTUs with >1% mean (across replicates) relative abundance in at least one sample. OTUs are arranged using complete-linkage clustering on pairwise distances according to Kimura's 2-parameter model. Figure for screen-view only!

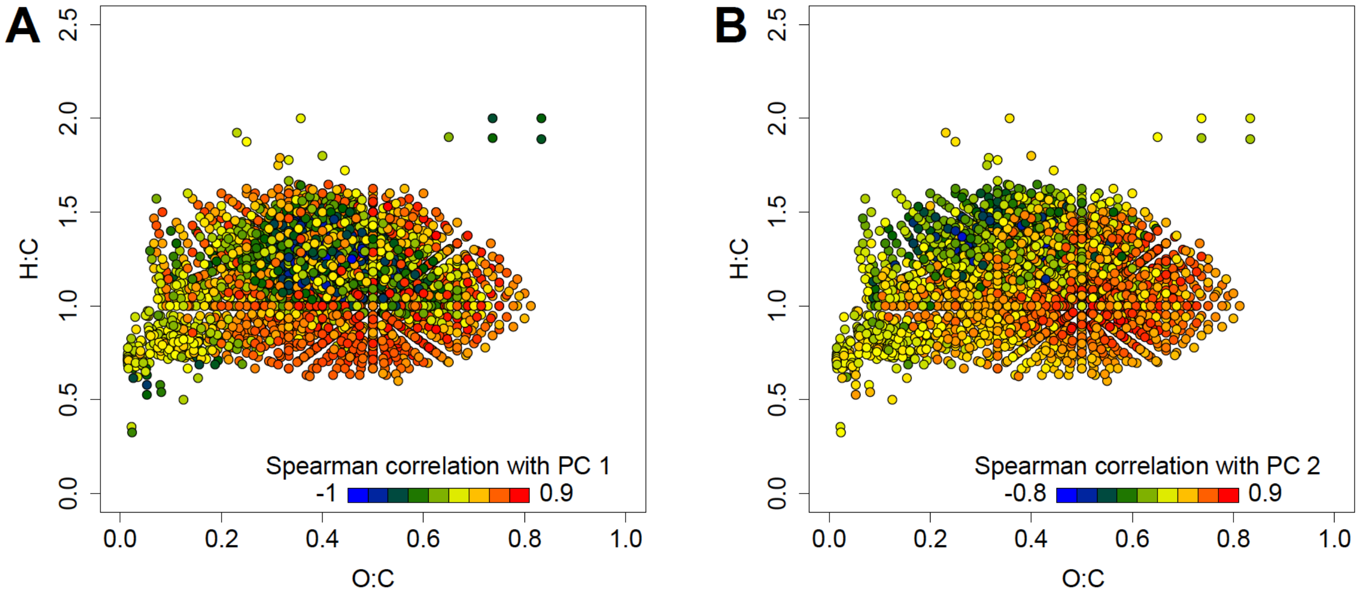

Figure S10. Main gradients of DOM composition in all well and bottled water samples at day 1 after filling. Van Krevelen plots of sum formulas identified by FT-ICR-MS. Each dot represents one sum formula and its location informs about oxygen richness and saturation. Dot color shows Spearman correlation coefficients of relative molecule intensity with the PC 1 **(A)** and the PC 2 **(B)**, which are the two dominant gradients of compositional variation (Figure 3). Only sum formulas detected at least 4 times are shown to avoid artificially inflated correlations.
